# Percutaneous Two-Dimensional Shear Wave Elastography for Diagnosis of Pancreatic Tumor

**DOI:** 10.3390/diagnostics11030498

**Published:** 2021-03-11

**Authors:** Yotaro Iino, Hitoshi Maruyama, Rintaro Mikata, Shin Yasui, Keisuke Koroki, Hiroki Nagashima, Masami Awatsu, Ayako Shingyoji, Yuko Kusakabe, Kazufumi Kobayashi, Soichiro Kiyono, Masato Nakamura, Hiroshi Ohyama, Harutoshi Sugiyama, Yuji Sakai, Tetsuhiro Chiba, Jun Kato, Toshio Tsuyuguchi, Naoya Kato

**Affiliations:** Department of Gastroenterology, Chiba University Graduate School of Medicine, 1-8-1 Inohan, Chuo-ku Chiba City 260-8670, Japan; homerun_amb@yahoo.co.jp (Y.I.); mikata@faculty.chiba-u.jp (R.M.); yasui_s@chiba-u.jp (S.Y.); koroki-keisuke@chiba-u.jp (K.K.); h.nagasima1120@chiba-u.jp (H.N.); afha7456@chiba-u.jp (M.A.); ayako0104maruno@gmail.com (A.S.); kusakabe.y@chiba-u.jp (Y.K.); kobayashi-kazufumi@chiba-u.jp (K.K.); kiyonosouichirou@yahoo.co.jp (S.K.); nkmr.chiba@gmail.com (M.N.); h_ohyama227@yahoo.co.jp (H.O.); sugiharu_food@chiba-u.jp (H.S.); sakai4754@yahoo.co.jp (Y.S.); techiba@faculty.chiba-u.jp (T.C.); kato.jun@chiba-u.jp (J.K.); tsuyuguchi-gi@umin.ac.jp (T.T.); kato.naoya@chiba-u.jp (N.K.)

**Keywords:** pancreatic cancer, 2D-shear wave elastography, ultrasonography, tumor-forming pancreatitis

## Abstract

Background: To investigate the efficacy of two-dimensional shear wave elastography (2D-SWE) for the diagnosis of pancreatic mass lesions. Methods: This ethics committee–approved cross-sectional study included 52 patients with histologically-proven pancreatic tumors (pancreatic ductal adenocarcinoma (PDAC), 36; tumor-forming pancreatitis (TFP), 15; neuroendocrine tumor, 1) and 33 control subjects. The 2D-SWE was performed for the tumor/non-tumor tissues, and SWE-mapping patterns and propagation quality were assessed. Results: Three mapping patterns were detected based on the size and distribution of the coloring areas. Pattern A (whole coloring) was detected in all non-tumor tissues and TFP, whereas pattern C (multiple small coloring spots) was detected in PDAC only. Pattern B (partial coloring with smaller spots) was detected in other lesions. The specificity and positive predictive value of pattern A for non-PDAC and those of pattern C for PDAC were 100%. The SWE value was higher in tumor lesions than in the non-tumor tissues (38.1 vs. 9.8 kPa; *p* < 0.001) in patients with PDAC. The SWE value in the non-tumor lesion was higher in patients with PDAC than in control (9.8 vs. 7.5 kPa; *p* < 0.001). Conclusions: 2D-SWE may play a role as a novel diagnostic tool for PDAC to detect a specific mapping pattern with quantitative assessment.

## 1. Introduction

Unfortunately, the global incidence of pancreatic ductal adenocarcinoma (PDAC) is increasing by about 1% annually [1]. Despite the advancement in the quality of medical care, patients with PDAC continue to experience unfavorable prognosis [2]. Presently, PDAC is the seventh leading cause of cancer-related mortality in the world, with more than 300,000 deaths reported each year [3].

Typically, imaging assessment is the initial process in the diagnosis of PDAC. Endoscopic ultrasonography (EUS) and/or endoscopic retrograde pancreatography may be considered as reliable tools with higher diagnostic ability [4,5,6]. However, because of the recent trend of reducing patient burden while ensuring safety, less-invasive methods may be preferred for practical management.

Shear wave elastography (SWE) is a novel, ultrasound (US)-based diagnostic technique used for the assessment of tissue elasticity in the liver, spleen, thyroid, and lymph nodes [7,8,9,10]. Of the several assessment methods, including strain imaging and impulse-based imaging [11], two-dimensional SWE (2D-SWE) has become rapidly popular because of its improved diagnostic performance with visible propagation quality [12,13]. Furthermore, a more recent study suggests that the use of propagation display method in SWE has the possibility to improve the reproducibility of measurement of pancreatic elastic modulus [14].

There are some studies on elasticity-related diagnosis of PDAC by using point SWE, such as virtual touch quantification (VTQ) or acoustic radiation force impulse (ARFI) [15,16]. Although investigators have reported a higher tendency of elasticity in pancreatic cancer lesions, the values are not statistically significantly elevated and there is an overlap between cancer and other diseases such as chronic pancreatitis or neuroendocrine tumor (NET) [17], which may be attributed to the heterogeneous tissue structure of the pancreatic tumor area. Therefore, the efficacy of elasticity measurement in the diagnosis of PDAC remains debatable.

Against this background, there is a possibility that the pattern of percutaneous 2D-SWE mapping may have the potential to characterize the tissue structural feature of PDAC heterogeneously typified with ill-defined masses and multinodular fashion [18]. Therefore, we have designed this prospective cross-sectional study to examine the effect of percutaneous 2D-SWE in the diagnosis of PDAC. This study aimed to investigate the specific findings on percutaneous 2D-SWE mapping images for the histologically proven PDAC as well as to provide the actual diagnostic performance.

## 2. Materials and Methods

### 2.1. Study and Patient Enrollment

This was a newly designed cross-sectional study conducted at Chiba university Hospital between August 2017 and February 2019. This study was performed in accordance with the Declaration of Helsinki after obtaining informed written consent from all study participants. This study protocol was approved by the Ethics Committee of Chiba University Hospital as having an appropriate design for publication.

This study prospectively recruited participants who met the following inclusion criteria:

- in-/out-patients with pancreatic tumor lesions and those scheduled for surgical procedure or fine needle biopsy (FNA) for histological examination or out-patients with focal pancreatic lesions that have already been confirmed histologically as benign by a past EUS-guided FNA (EUS-FNA); and
- patients without any abdominal surgical history. The final diagnosis was based on the histological analysis of surgically resected specimens or EUS-FNA results. The control group comprised participants lacking a history of pancreatic disease or abdominal symptoms. However, patients who were pregnant at the time of screening were excluded.

Percutaneous US and 2D-SWE examinations were scheduled for the study participants.

### 2.2. ERCP, EUS, and EUS-FNA

ERCP was performed with a duodenoscope (JF 260V; Olympus, Tokyo, Japan). Cannulation of the pancreatic duct or bile duct, which was depending on the case, was performed using a 1.7-mm-diameter cannula (PR-104Q; Olympus, Tokyo, Japan). At least two board certified fellows of the Japan gastroenterological endoscopy society evaluated the endoscopic cholangiograms or pancreatograms and made a diagnosis referring to the typical image of PDAC, which was an interrupted image of the main pancreatic duct, or the typical images of AIP, which were side branch arising from narrowed portion of the main pancreatic duct and skip lesions [19].

EUS and EUS-FNA was performed using a linear echoendoscope (GF-UCT260; Olympus, Tokyo, Japan) and the US observation apparatus (EU-ME2 PREMIER PLUS; Olympus, Tokyo, Japan) equipped with a 22- or 25-gauge needle (Acquire; Boston Scientific Japan, Tokyo, Japan). EUS criteria for PDAC included hypoechoic inhomogenous mass, with irregular margins [20]. Specimens obtained were classified into five categories: malignancy, suspected malignancy, atypical cells, no evidence of malignancy, or insufficient material. Specimens classified as malignancy or suspected malignancy was defined as malignant based on EUS-FNA results [21].

### 2.3. SWE

We used the Aplio 500 (Canon, Tokyo, Japan) with a 3.75-MHz convex probe in this study. Patients observed >12 h fasting and were examined in the supine position. First, the primary operator (HM), a board-certified physician with >25 years of experience in using US-based diagnostic examination performed B-mode US to detect the pancreas and pancreatic tumor lesions. Then, SWE was performed under appropriate plane (basically transverse scan) under breath-holding for a few seconds for the tumor lesion, followed by that for the non-tumor lesion, which was at the proximal side of the tumor. However, the SWE was performed only for the non-tumor lesions in the control subjects.

Repeated SWE was conducted (more than 10 times per individual, at least five times for tumor lesions and five times for non-tumor lesions) to confirm the reproducibility of the SWE-mapping patterns [14]. Next, the same procedure was performed by a second operator, with 6 years of experience in US-based diagnosis, in 21 randomly selected patients to analyze the inter-operator variability of the SWE findings. Intraclass correlation coefficients were obtained from valid measured SWE values.

### 2.4. SWE Data Assessment

**Figure 1:**
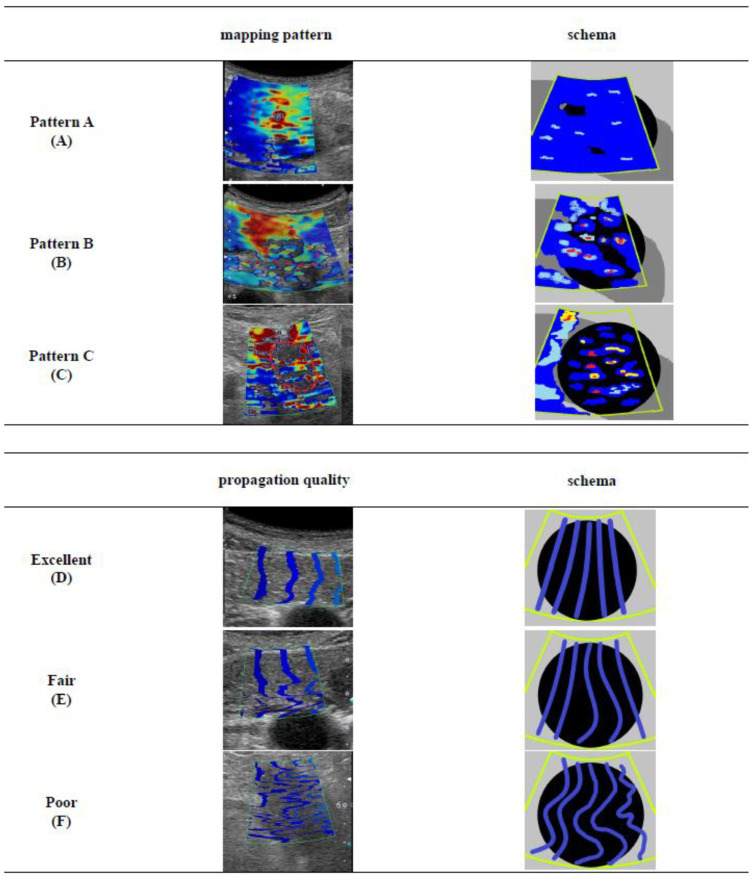
The definitions of shear wave elastography (SWE) mapping patterns and propagation qualities: (**A**) Mapping pattern A, whole coloring over the tumor/non-tumor lesion; (**B**) Mapping pattern B, presence of partial coloring area (major color-displayed area showing 50% or more of the target lesion) and small isolated coloring spots (major color-displayed area showing less than 50% of the target lesion) and uncolored in the other part; (**C**) Mapping pattern C, multiple small isolated coloring spots (major color-displayed area showing less than 50% of the target lesion) with uncolored areas; (**D**) Propagation quality “Excellent”, linear contour lines are with equally spaced intervals; (**E**) Propagation quality “Fair”, some of the lines are showing unequally spaced and/or non-linear appearance; (**F**) Propagation quality “Poor”, all lines are demonstrating a non-linear appearance with non-linear shaped lines.

The SWE-mapping patterns were classified into three patterns (Figure 1): (A) whole coloring over the tumor/non-tumor lesion; (B) presence of partial coloring area (larger than half of the target lesion, major color-displayed area in the target lesion ≥ 50% or more) and small isolated coloring spots (major color-displayed area in the target lesion < 50%) and uncolored areas in the other parts; and (C) multiple small isolated coloring spots with uncolored areas.

Propagation quality was evaluated using three classifications according to the literature [22]. Excellent propagation (D), with linear contour lines with equally spaced intervals; fair propagation (E), with some of the lines showing unequally spaced and/or non-linear appearance; and poor propagation (F), with all lines demonstrating a non-linear appearance with non-linear shaped lines. Excellent and fair propagation were determined as the acceptable quality.

The SWE-mapping patterns and the propagation quality were also assessed by the blind reading of two independent reviewers (SY and KK) to confirm the objectivity of the classification and determination of the classification.

The median of the SWE value (kilopascals, kPa) and standard deviation (SD) were obtained using multiple circular ROIs (4 or more/image) with the appropriate size set in the tumor (and non-tumor) lesion. Then, the SWE value and SD in tumor/non-tumor ratio was calculated.

### 2.5. Statistical Analysis

All statistical analyses were conducted using SPSS ver. 22 software (IBM-SPSS, Inc. Chicago, IL, USA). Fisher exact test or χ^2^ test was used for categorical variables, and the Mann–Whitney *U*-test was used for continuous variables, as deemed appropriate. The statistical significance was set at *p* < 0.05. The optimal cut-off value of the variables that differentiated between PDAC and non-PDAC in the mapping pattern B and the area under the curve was determined by the receiver-operating characteristic analysis.

## 3. Results

### 3.1. Characteristics of the Participants

**Figure 2:**
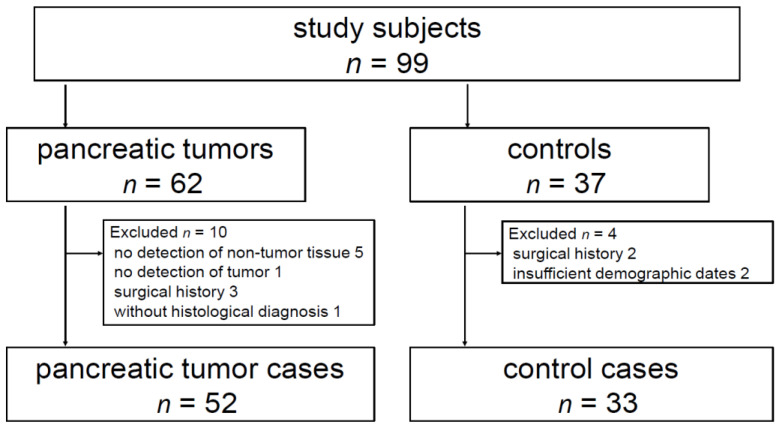
A total of 62 patients with pancreatic tumor lesions and 37 control subjects were screened. In the former group, 10 patients were not eligible for the inclusion criteria (5 patients whose non-tumor pancreatic parenchyma was not sufficiently detected for SWE measurement because of the presence of pancreatic head tumor, 3 patients with surgical history, 1 patient without historical diagnosis, and 1 patient whose pancreatic tumor was not clearly detected by B-mode ultrasound (US)). In the latter group, 2 cases had surgical history and 2 cases had no laboratory data. Finally, a total of 52 patients with pancreatic tumor lesions and 33 control subjects were included in the study.

A total of 62 patients with pancreatic tumor lesions and 37 control subjects were screened (Figure 2). In the former group, 10 patients were not eligible for the inclusion criteria (5 patients whose non-tumor pancreatic parenchyma was not sufficiently detected for SWE measurement because of the presence of pancreatic head tumor, 3 patients with surgical history, 1 patient without historical diagnosis, and 1 patient whose pancreatic tumor was not clearly detected by B-mode US. In the latter group, 2 cases had surgical history and 2 cases had no laboratory data. Finally, a total of 52 patients with pancreatic tumor lesions and 33 control subjects were included in the study (Table 1). The location of the pancreatic tumor was the head in 22 patients (PDAC, 13; non-PDAC, 9), body in 23 (PDAC, 16; non-PDAC, 7), and tail in 7 (PDAC, 7), with a maximum diameter of 13.0–69.0 mm (mean: 30.0 ± 12.3 mm) for PDAC and 14.0–40.0 mm (mean: 20.3 ± 8.25 mm) for non-PDAC. The US pattern in the tumor lesion was hypoechoic in 48 patients and isoechoic in four patients.

The histological diagnosis was made by surgical specimens in 14 patients and by FNA in 38 patients, resulting in the diagnosis of adenocarcinoma in 36 patients, NET in 1, and tumor-forming pancreatitis (TFP) in 15 (autoimmune pancreatitis (AIP), 12; chronic pancreatitis, 3). The staging of PDAC, which was based on the tumor nodule metastasis system, was stage IA in one patient, stage II in 17 patients, stage III in 5, and stage IV in 13.

ERCP was performed for the diagnosis of pancreatic tumors in 12 patients in whom informed consent was obtained. Also, EUS was performed in all patients with pancreatic lesions. The diagnostic performance of ERCP and EUS was 100% and 100% for sensitivity, 42.9% and 68.8% for specificity, and 55.6% and 87.8% positive predictive value (PPV), respectively.

### 3.2. SWE-Mapping Patterns and Propagation Quality

The SWE procedures were successfully performed in all patients with pancreatic tumor lesions and in all control subjects. The non-tumor pancreatic tissue showed pattern A in all control subjects and in 52 patients with pancreatic tumor lesions. Meanwhile, three mapping patterns were noted in the pancreatic tumor lesions: pattern A in 3 patients, B in 24, and C in 25 (Table 2).

The inter-operator agreement in the SWE-mapping patterns was 100% (21/21). There were not any differences in the reproducibility in the cases of pattern A, pattern B and pattern C. The intraclass correlation coefficients for SWE value was 0.8480. The blind reading between two independent reviewers revealed an agreement rate of 96.2% in the mapping pattern classification and 86.5% in the propagation quality.

The propagation quality was acceptable in all control subjects (excellent in 22 subjects and fair in 11 subjects) and that of non-tumor pancreatic tissue was acceptable in 52 patients (excellent in 30 patients and fair in 22 patients). Acceptable quality was significantly more frequent in pattern A tumor lesions (3/3, 100%) than in pattern C lesions (7/25, 28.0%; *p* < 0.001). Meanwhile, in patients with pattern C pancreatic tumor lesions, although poor quality was detected in 32 of the 52 tumor lesions (61.5%), acceptable quality was found to be dominant in non-tumor pancreatic tissues in 52 of the 52 tumor lesions (100%).

### 3.3. Diagnostic Performance of SWE Findings for Pancreatic Tumors

Pattern A was detected in all control subjects and in 3 patients with TFP, whereas pattern C was detected in only patients with PDAC. Pattern B was detected in various types of pancreatic lesions: 11 in PDAC and 13 in non-PDAC (TFP, 12; NET, 1).

**Figure 3:**
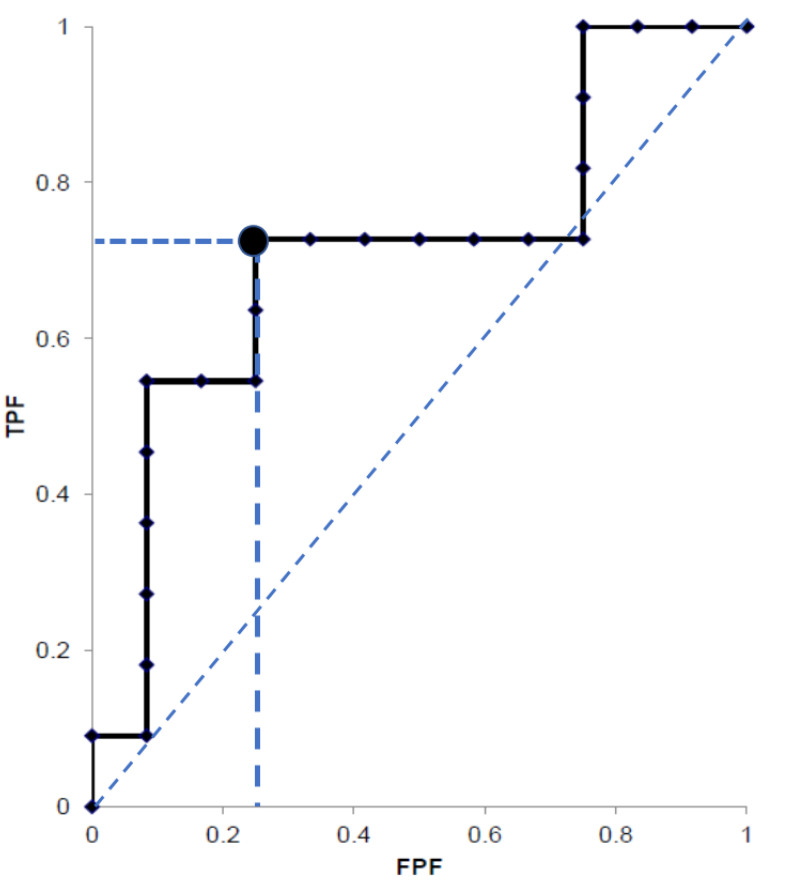
The best cut-off value of SWE tumor/non-tumor ratio was 2.42 to identify PDAC showing 0.7692 AUROC in the pancreatic tumors showing pattern B. TPF, true positive fraction; FPF, false positive fraction.

**Figure 4:**
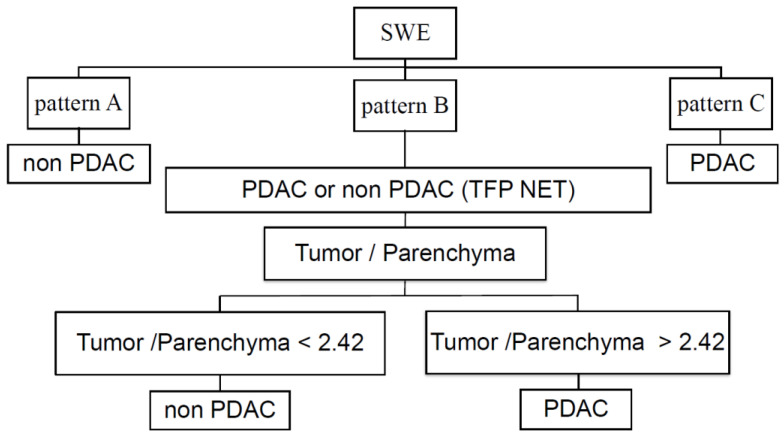
Diagnostic algorithm of pancreatic lesion was proposed by using SWE mapping patterns and SWE value ratio between tumor and non-tumor tissue.

The SWE value was significantly higher in tumor lesions (38.1; IQR 19.4–62.2 kPa) than in the non-tumor tissue (9.8; IQR 8.7–11.6 kPa; *p* < 0.001) in all patients with PDAC. The SWE tumor/non-tumor ratio was higher in the PDAC (*n* = 36; 3.59; IQR 2.43–5.47 kPa) than in the non-PDAC tumors (*n* = 16; 1.48; IQR 1.10–1.83; *p* < 0.001). In addition, in pancreatic tumors with pattern B, although there were no differences in the characteristics between the PDAC and non-PDAC (Table 3), the SWE tumor/non-tumor ratio was higher in PDAC (3.89, IQR 2.37–5.17) than in non-PDAC lesions (1.53, IQR 1.12–1.78 kPa; *p* = 0.0045 [NET, 1; PDAC vs. benign *p* < 0.001]). Sensitivity, specificity, positive predictive value (PPV), negative predictive value (NPV), and the accuracy of pattern A to diagnose non-PDAC lesions were 18.8%, 100%, 100%, 73.5%, and 75.0%, respectively, and those of pattern C to diagnose PDAC were 66.7%, 100%, 100%, 57.1%, and 76.9%, respectively. In the tumors showing pattern B, the best cut-off value of SWE tumor/non-tumor ratio was 2.42 to identify PDAC, with 72.7% sensitivity, 76.9% specificity, 75.9% PPV, 73.8% NPV, 74.9% accuracy, and 0.7692 AUROC (Figure 3). From these results of SWE mapping patterns and SWE values, we proposed the novel diagnostic algorithm for pancreatic tumor (Figure 4). If the data was assessed only in 37 patients with pancreatic neoplasms, the diagnostic performance of pattern C for pancreatic cancer was 69.4% sensitivity, 100% specificity, and 100% PPV. The SWE value of the non-tumor tissues in patients with PDAC was significantly higher than that in control subjects (9.8; IQR 8.7–11.6 vs. 7.5; IQR 6.9–8.7; *p* < 0.001).

## 4. Discussion

The present study clearly demonstrated a novel aspect of applying percutaneous 2D-SWE for the diagnosis of PDAC. The three mapping patterns detected in the pancreatic tumor lesions were easily distinguishable and supported by the objective judgment with high inter-operator agreement and blind review results. Pattern C was specific to PDAC with the diagnostic performance revealing high specificity and PPV. The close relationship between pattern C and PDAC may be explained by the irregular acoustic propagation caused by flection and refraction due to the cancer-related intra-tumor heterogeneous structure. This observation is supported by the high incidence of poor propagation quality in the tumor lesions, but not in the non-tumor pancreatic tissues.

The present study demonstrated a close linkage between SWE mapping pattern and propagation quality; pattern A linked with acceptable propagation quality and pattern C linked with poor propagation quality. There may be an argument against the validity of pattern C showing heterogeneous coloring and/or presence of defect, because poor propagation quality may suggest that the SWE data is less reliable [22]. Actually, it is reported that a degree of fibrosis and presence of ascites appear to be independent to determine the propagation quality and only BMI showed close linkage [22]. That is, the “quality” may be affected by the inner factor like structural appearance of the target as well as the outer factor such as patient condition and operator’s technique. Based on the sufficient reproducibility and high inter-reviewer agreement in the present study, however, the authors emphasize that pattern C is according to an unavoidable poor propagation due to cancer-related tumor appearance, but not related with the low reliability. That is, the role of color mapping could be a visualization of tissue structure based on acoustic propagation. However, obviously, it needs to be further validated in the larger patient population.

The patterns A/B were detected in non-tumor pancreatic tissues and various pancreatic lesions, including TFP (AIP dominant), NET, and PDAC. The AIP is characterized by the histological findings of periductal lymphoplasmacytic infiltration and storiform fibrosis [23]. The NET represents a broad and heterogeneous group of neoplasms with diverse clinical and pathological characteristics, showing wide morphological repertoire, including oncocytic, pleomorphic, ductulo-insular, sclerosing, and lipid-rich variants [24]. The degree of these pathological abnormalities may account for the different mapping patterns. However, in the present study, we did not perform macroscopic histological investigation in all patients because some of the cases were diagnosed using FNA-based small samples alone. Further study may be needed to examine the substantial relationship between the histological structural findings and SWE-mapping patterns.

Our study demonstrated the SWE tumor/non-tumor ratio of 2.42 as the best cut-off value to differentiate PDAC from non-PDAC pancreatic lesions in patients with pattern B. The data is supported by the VTQ-related study that reported higher mean shear wave velocity (SWV) of the pancreas harboring pancreatic cancer as compared with that of the pancreas without cancer (1.51 ± 0.45 m/s vs. 1.43 ± 0.28 m/s), although they did not reach statistical significance [25]. 

As for the diagnostic performance, a more recent study using VTQ revealed that sensitivity, specificity, PPV, and NPV for PDAC were 88%, 73%, 51%, and 95%, respectively, by the cut-off values of SWV 1.40 m/s compared with pancreatic parenchyma in patients with PDAC [15]. Moreover, ARFI has resulted in 91.1% sensitivity and 60.4% specificity with 78.6% AUROC for pancreatic malignant disease under a cut-off value of 1.74 m/s, and ARFI value >3.95 m/s was detected in only malignancy [17]. The diagnostic performance of the present study appeared comparable to those reported previously, which may be explained by the advantage of our technique with the possible visualization of the whole cross-section images, not limited area by point SWE using VTQ/ARFI. Interestingly, our study also demonstrated higher SWE value of non-tumor pancreatic tissues in patients with pancreatic lesions than in control subjects, and similar data have been reported by Kawada et al., although they failed to detect any significant difference [25]. This result may be explained by the influence caused by tumor-related inflammation. However, it may also suggest the potential difference in the pancreatic parenchyma being linked to carcinogenesis, which remains to be elucidated.

EUS is a popular and reliable tool to characterize pancreatic tumors, and the recent developments have introduced a built-in elastography technique [26,27,28]. There are two ways to evaluate the findings: qualitative assessment of mapping pattern and quantitative assessment of the strain ratio. For the former, the study using dominant color mapping analysis revealed 100% sensitivity, 85.5% specificity, 90.7% PPV, 100% NPV, and 94.0% overall accuracy for the diagnosis of malignancy [29]. Another multicenter study proposed a classification system using a scoring system and reported sensitivity and specificity of EUS elastography to differentiate benign from malignant pancreatic lesions of 92.3% and 80.0%, respectively, in comparison to 92.3% and 68.9%, respectively, for the conventional B-mode images [30]. Meanwhile, for the latter, a Spanish study reported that the strain ratio using the soft reference area for detecting pancreatic malignancies revealed 100% sensitivity and 92.9% specificity with 0.983 AUROC [31]. According to the study from United Kingdom, the diagnostic abilities for discriminating malignant from benign pancreatic masses were 100% sensitivity, 16.7% specificity, 86.1% PPV, 100% NPV, and 86.5% overall accuracy [32]. Cumulatively, the diagnostic ability depends on the manner of analysis, including the reference area, and the relatively lower specificity may limit the value of this technique with possible complications, and transabdominal 2D-SWE may have a competitive advantage in this regard.

Endoscopic technique using ERCP or EUS may have an important role to diagnose pancreatic diseases [33]. However, as a nature of endoscopic procedure, invasiveness is highly problematic [34] and relatively low specificity presented in our data may limit the practical value. It is strongly suggested the 2D-SWE may be much easier and safer to perform than endoscopic methods and more affordable for patients.

There are some limitations to our study. First, the sample size is relatively small, particularly with a small number of benign pancreatic lesions and the number of registered cases of NETs was only one. Next, the physical size with BMI from 15.5 to 31.2 kg/m^2^ may indicate favorable conditions for US observation in comparison with that among Western patients. Furthermore, the stage ranged from II to IV in the majority of PDAC and there was only one case with stage I. Additional studies with larger patient cohorts, including various types of pancreatic diseases, are warranted to validate our data and to clarify the benefit of our technique for early detection of PDAC.

## 5. Conclusions

In conclusion, percutaneous 2D-SWE may play a role as a novel diagnostic tool for PDAC to detect a specific pattern with quantitative assessment. Particularly, EUS-FNA might be unnecessary in patients with SWE pattern C. The application of this technique is strongly recommended for focal lesions in the pancreas, because it can facilitate definitive malignant decision without any invasiveness.

## Figures and Tables

**Table 1:** Patient characteristics.

Characteristics	PDAC (*n* = 36)	Non-PDAC (*n* = 16)		Control
		TFP (*n* = 15)	NET (*n* = 1)	(*n* = 33)
Sex (Male/Female)	22/14	10/5	1/0	12/21
Age (year)	68.3 ± 12.2	64.0 ± 10.4	64	68.0 ± 12.4
	(35–87)	(48–84)	-	(35-94)
BMI (kg/m^2^)	21.4 ± 3.19	22.3 ± 3.95	24.6	22.4 ± 2.46
	(15.5–28.5)	(17.0–31.2)	-	(18.3-28.3
Smoking, *n* (%)	18 (50)	6 (40)	0 (0)	12(36.4)
Alcohol, *n* (%)	4 (11.1)	3 (20)	0 (0)	3(9.1)
Diabetes mellitus, *n* (%)	8 (22.2)	5 (33.3)	0 (0)	4(12.1)
Pancreatic lesion				
Location (Head/Body or Tail)	13/23	8/7	1/0	
Size (mm)	28.0 ± 12.3	28.6 ± 8.86	22.0	
	(13.0–69.6)	(14.0–40.0)	-	
Histological assessment				
(FNA/Resection)	23/13	15/0	0/1	

PDAC, pancreatic ductal adenocarcinoma; TFP, tumor-forming pancreatitis; NET, neuroendocrine tumor; BMI, body mass index; FNA, fine needle aspiration.

**Table 2:** SWE mapping patterns and patient characteristics.

Characteristics	Mapping Pattern		
	A	B	C
Number (case)	3	24	25
PDAC/TFP/ NET	0/3/0	11/12/1	25/0/0
Age (year)	64.7 ± 10.7	65.0 ± 11.3	69.2 ± 12.1
	(53–74)	(44–87)	(35–85)
BMI (kg/m^2^)	21.9 ± 5.59	22.9 ± 3.65	20.8 ± 3.04
	(17.0–28.0)	(17.0–31.1)	(15.5–28.5)
Smoking, *n* (%)	1 (33.3)	9 (37.5)	14 (56.0)
Alcohol, *n* (%)	0	4 (16.7)	3 (12.0)
Pancreatic lesion			
Size (mm)	20.4 ± 7.88	26.2 ± 6.95	32.5 ± 14.0
	(14.0–29.9)	(15.0–40.0)	(13.0–69.0)
Location (head), *n* (%)	1 (33.3)	13 (54.2)	8 (32.0)
SWE (kPa)	10.6	22.1	27.1
IQR	(7.3–11.3)	(13.0–56.2)	(13.0–148.3)
SD (kPa)	4.94	17.6	27.1
IQR	(3.6–5.7)	(9.5–27.7)	(13.0–48.3)
SWE tumor/non-tumor ratio	1.21	1.97	3.37
IQR	(1.07–1.60)	(1.43–4.07)	(2.47–5.46)

SWE, shear wave elastography; PDAC, pancreatic ductal adenocarcinoma; TFP, tumor-forming pancreatitis; NET, neuroendocrine tumor; BMI, body mass index; SD, standard deviation.

**Table 3:** Patients characteristics with mapping pattern B.

Characteristics	PDAC	Non-PDAC	*p*-Value
Number	11	13	
Age (year)	66.3 ± 12.8	65 ± 10.4	*p* = 0.5236
	(44–87)	(48–84)	
BMI (kg/m^2^)	22.7 ± 3.29	23.1 ± 4.14	*p* = 0.8696
	(17.0–27.8)	(18.9–31.2)	
Diabetes mellitus, *n* (%)	2 (18.2)	5 (38.5)	*p* = 0.6576
Smoking, *n* (%)	4 (36.4)	4 (30.8)	*p* = 0.8190
Alcohol, *n* (%)	1 (9.1)	3 (23.1)	*p* = 0.5207
Lesion			
SWE value (kPa)	34.7	26.2	*p* = 0.0987
IQR	(23.1–69.1)	(12.8–44.6)	
SD (kPa)	25.6	17.0	*p* = 0.2466
IQR	(12.2–29.9)	(6.93–25.3)	
kPa/SD	1.58	1.28	*p* = 0.7721
IQR	(1.17–1.97)	(1.12–2.28)	
Parenchyma			
SWE value (kPa)	10.2	9.86	*p* = 0.6021
IQR	(9.8–12.9)	(9.22–15.1)	
SD (kPa)	3.38	5.83	*p* = 0.1320
IQR	(2.49–5.48)	(4.10–6.90)	
kPa/SD	3.57	1.80	*p* = 0.2024
IQR	(1.82–5.86)	(1.12–2.28)	
SWE tumor/non-tumor ratio			
SWE value (kPa)	3.89	1.53	*p* = 0.0045
IQR	(2.37–5.17)	(1.12–1.78)	

PDAC, pancreatic ductal adenocarcinoma; BMI, body mass index.
